# Influence of Mix Design on Physical, Mechanical and Durability Properties of Multi-Recycled Aggregate Concrete

**DOI:** 10.3390/ma16072744

**Published:** 2023-03-29

**Authors:** Jeonghyun Kim, Anna M. Grabiec, Andrzej Ubysz, Sungchul Yang, Namho Kim

**Affiliations:** 1 Faculty of Civil Engineering, Wrocław University of Science and Technology, Wybrzeże Wyspiańskiego 27, 50-370 Wrocław, Poland; 2 Faculty of Environmental Engineering and Mechanical Engineering, Poznań University of Life Sciences, 60-649 Poznań, Poland; 3 School of Architectural Engineering, Hongik University, 2639 Sejong-ro, Jochiwon, Sejong 30016, Republic of Korea; 4 School of Industrial Design & Architectural Engineering, Korea University of Technology & Education, 1600 Chungjeol-ro, Byeongcheon-myeon, Dongnam-gu, Cheonan 31253, Republic of Korea

**Keywords:** true sustainability, repeated recycling, construction and demolition waste, concrete mix design, recycled concrete aggregate, green construction

## Abstract

The decrease in the quality of recycled aggregate due to an increase in the number of recycling is a primary factor that limits the multi-recycling of concrete. This degradation adversely affects concrete performance; thus, the characteristics of recycled aggregate should be considered during the mix design stage, but little research has taken that into account. This study investigates the effect of the equivalent mortar volume (EMV) mix design on some physical, mechanical and durability properties of concrete made of multiple recycled coarse aggregates at 50% and 100% replacement ratios compared to concrete made by the conventional mix design (CMD). The results showed that the performances of concrete by the CMD decreased with an increasing number of recycling cycles. The properties of EMV-based concrete deteriorated with an increase in the number of recycling cycles at 100% replacement ratio due to poor workability caused by a shortage of fresh mortar. However, at 50% replacement, the EMV-based concrete exhibited similar performance across the three cycles of recycling, as well as improved properties over natural aggregate concrete. This study demonstrated that an appropriate mix design and optimal aggregate replacement ratio can offset the property loss of multiple recycled aggregate concrete.

## 1. Introduction

Extensive and diverse studies on recycled aggregate for green construction have led to the establishment of various technical guidelines and standards for its use in many countries and institutes [1,2,3,4,5,6]. In general, recycled aggregate, particularly of coarse size obtained from concrete waste, is produced by crushing concrete made with natural aggregate in various ways. Concrete containing the recycled aggregate as raw material is named recycled aggregate concrete (RAC). Technically, this recycled aggregate is ‘recycled aggregate used once’. However, in order to achieve sustainability in a true sense, a discussion of multiple recycling of recycled aggregate and RAC needs to be conducted.

To date, studies on multiple recycled aggregate concrete (MRAC) have been conducted by a limited number of scholars, but this topic is intriguing to many researchers, and the interest is clearly growing. In general, the multiple recycling of concrete progressively reduces its mechanical properties in proportion to the number of recycling cycles. In a study by Abreu et al. [7], the 28-day compressive strength of first, second, and third generation RAC decreased by 3.2%, 4.7%, and 13.1%, respectively, and the tensile strength decreased by 9.1%, 11.4%, and 15.1%, compared to natural aggregate concrete (NAC). Huda and Alam [8] also reported that the 28-day compressive strength and tensile strength of third generation RAC decreased by about 40% and 33% of those of NAC. Moreover, the third generation RAC required 120-day curing to achieve compressive strength similar to that of NAC. The multiple recycling also has unfavorable influences on the durability of concrete, such as frost, chloride and carbonation resistance [9]. In a study by Zhu et al. [10], third generation RAC was able to withstand only 600 cycles of freeze-thaw action unlike NAC and first and second generation RACs, which showed a relative dynamic modulus of approximately 55–65% after 800 freeze–thaw cycles. In addition, the chloride ion permeability coefficients of first, second and third generation RACs were 39.6%, 59.8%, and 84.9% higher than that of NAC.

The aforementioned studies have investigated the effect of multiple recycling of concrete and reported the progressive decrease in performance as the number of times of recycling increases [11,12,13]. As a rule, the quality degradation of recycled aggregate occurring in the repeated recycling is identified as the cause of the gradual decrease in MRAC performance [14]. It is a well-known fact that the quality of recycled aggregate is one of the parameters that govern the performance of RAC. The adhered mortar of recycled aggregate increases the total mortar volume of RAC and weakens the interfacial transition zone (ITZ) between aggregate and cement matrix, which is responsible for reducing performance. Thomas et al. [15] reported that third generation RAC has almost twice as much mortar content as first generation RAC, which limits the repeated recycling of concrete. In this context, it appears that there are certain limitations associated with the repeated recycling of concrete. However, no efforts have been made to address the performance degradation that occurs as a result of its multiple recycling. Hence, there is a need to conduct investigations aimed at finding solutions that can enhance the performance of MRAC, and the solutions should be as energy-friendly as possible, taking into account global trends towards the environment. At this point, the application of the equivalent mortar volume (EMV) method can be considered to offset the performance losses of the RAC caused by the degraded quality of the recycled aggregate. The EMV method is a type of mix design that maintains the volume of total mortar in RAC (i.e., the adhered mortar in recycled aggregate and fresh mortar) constant by considering the adhered mortar in recycled aggregate as mortar, not aggregate. Not only the mechanical and durability performance of the EMV-based concrete has been proven in many previous studies, but the EMV method is also considered to be eco-friendly and economical as it can save the amount of fresh mortar as much as the volume of adhered mortar of recycled aggregate [16,17,18,19,20]. In particular, the EMV method has been reported to be effective even for low-quality aggregates [21], which can be expected to contribute to enhancing the performance of MRAC by reducing the negative influence of the quality degradation of recycled aggregate caused by multiple recycling.

Therefore, this study aims to enhance the performance of MRAC. A series of MRACs containing once-, twice- and thrice-recycled coarse aggregates (RCA) were prepared by the conventional mix design (CMD) and the EMV methods, respectively, and their physical, mechanical and durability properties were analyzed. The obtained experimental data are expected to make an important contribution to complementing the lack of knowledge about MRAC. After all, the ultimate goal of this study is to explore true sustainability of concrete through multiple recycling.

## 2. Experimental Program

### 2.1. Research Flow

**Figure 1:**
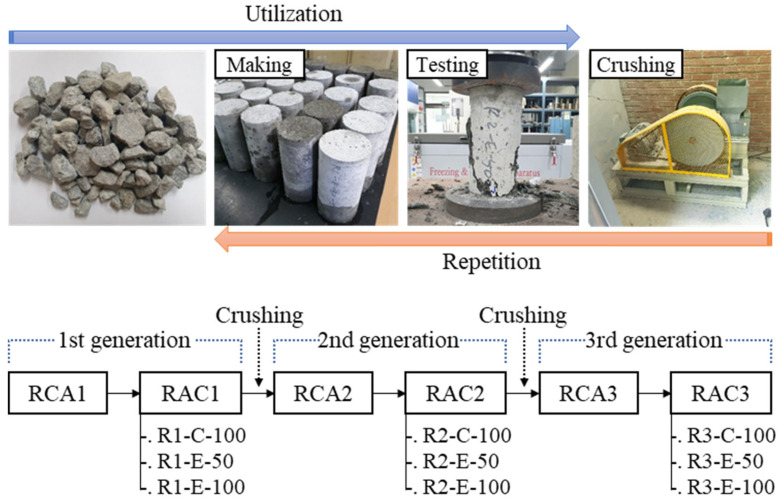
Experimental flow of multiple recycled aggregate concrete.

The experimental procedure of this study is shown in Figure 1. The experiment involved a series of concrete making and crushing processes to prepare first, second and third generation RCAs and RACs, which is then subjected to tests to evaluate their physical, mechanical and durability properties. The RCAs and RACs during three recycling cycles were defined as follows:

- RCA1: it refers to the first generation RCA produced by multiple crushing precast concrete members made of natural aggregate in a recycling plant.
- RAC1: the first generation RAC containing RCA1 is denoted as RAC1, and it is subdivided into R1-C-100, R1-E-50, and R1-E-100 according to the mix design and replacement ratio of RCA1.
- RCA2: the second generation RCA, RCA2, was obtained by crushing RAC1 with 100% RCA1 (i.e., R1-C-100 and R1-E-100) with a laboratory jaw crusher after 35 days of curing. In particular, R1-E-50, which contains 50% of natural coarse aggregate (NCA), was excluded from RCA2 production in order to ensure the representativeness of being recycled twice.
- RAC2: the second generation concrete uses RCA2, and it is divided into R2-C-100, R2-E-50, and R2-E-100 based on the mix design and RCA2 replacement ratio.
- RCA3: this refers to RCA obtained from RAC2 with 100% RCA2 (i.e., R2-C-100 and R2-E-100). For the same reason as in the RCA2 preparation described above, R2-E-50 was excluded from RCA3 production.
- RAC3: the third generation RAC. RAC3 was produced using RCA3 as a coarse aggregate, and it is classified into R3-C-100, R3-E-50, and R3-E-100 depending on the mix design and aggregate replacement ratio.

### 2.2. Materials

**Figure 2:**
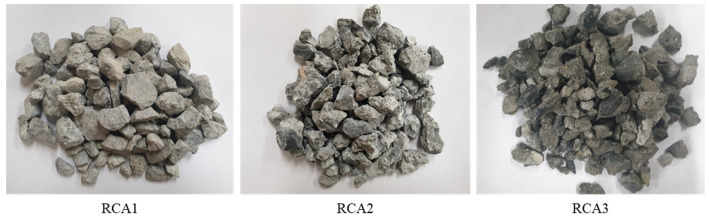
Recycled concrete aggregates with different recycling generations.

**Figure 3:**
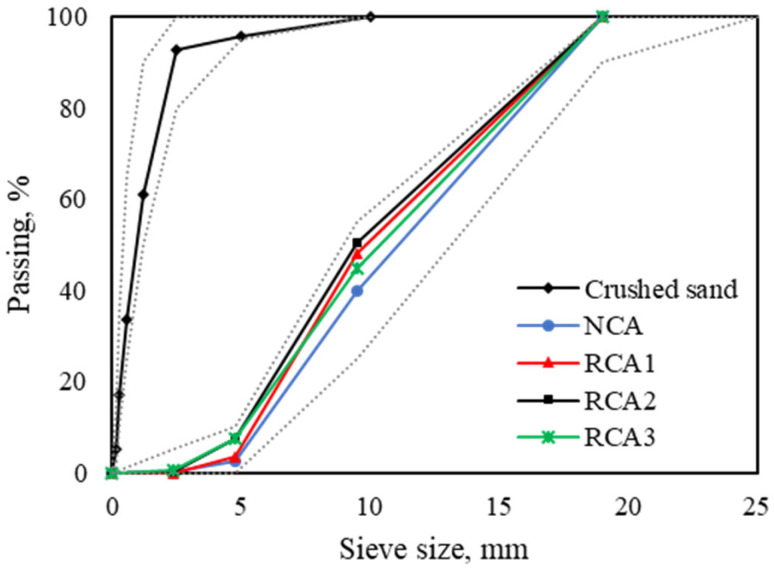
Particle size distributions of aggregates.

In this experimental research program, ordinary Portland cement was used as a binder, and its properties are provided in Table 1. For the fine aggregate, crushed sand with a nominal maximum size of 4.75 mm was used, and the water absorption and specific gravity were 1.09% and 2.59, respectively. For coarse aggregate, granite natural aggregate and three different generations of RCA (RCA1, RCA2, RCA3) were used (Figure 2), and the size fraction of 4.75–19 mm was selected through sieving. The particle size distribution and physical characteristics of the aggregates used are given in Figure 3 and Table 2.

### 2.3. Mixture Design

In order to investigate the mechanical and durability properties of MRAC, a total of 10 concrete mixtures were designed by the CMD and the EMV design [22]: reference concrete, 3 series of RAC1, 3 series of RAC2, 3 series of RAC3.

The mix proportion of each mixture is detailed in Table 3. The CMD does not consider the variation of RCA characteristics caused by multiple recycling. Therefore, the CMD-based concretes (R1-C, R2-C and R3-C in Table 3) were designed with a constant amount of cement, sand and water required to make unit concrete. In contrast, with the EMV method, the mix proportion of concrete varied depending on the adhered mortar content in RCA. The volume of fresh mortar in EMV-based concrete was deducted by the volume of hardened mortar adhered to RCA, so that the EMV-based concrete had a constant aggregate–mortar ratio regardless of the amount of adhered mortar. As the adhesive mortar content of RCA increased with the number of recycling cycles, the amount of cement, sand, and water required for unit concrete decreased in the order of R1, R2, and R3. A detailed description of the mix proportions of concrete designed with the EMV method can be found in our previous studies [23,24].

- Reference concrete was prepared using natural aggregates, and the mix proportion provided by a ready-mixed concrete plant was adopted, which was set to achieve a 28-day compressive strength of 30 MPa and a slump of 180 mm.
- For RAC1, two mix designs were used: CMD and EMV. Concrete made by CMD was named R1-C, and the concrete made with the EMV mix design was divided into R1-E-50 and R1-E-100 based on the RCA1 replacement ratio.
- The RAC2 with the CMD was named R2-C, and RAC2 proportioned by the EMV design was divided into R2-E-50 and R2-E-100 depending on the RCA2 replacement ratio. Particularly, at 100% replacement ratio, the combination of RCA2 with adhered mortar content of 23% and the EMV design significantly reduced the amount of fresh mortar, making it nearly impossible to mold with the general compaction (not only zero slump, but hard to compact by steel rod), thus the modified EMV design proposed by Yang and Lee [25] was applied. The modified EMV method considers only a certain portion of the adhered mortar content of RCA as mortar and the remaining portion as aggregate, thereby increasing the amount of fresh mortar. In practice, the determination of the portion of mortar and aggregate in adhered mortar depends on the experience and knowledge of concrete designer. In this study, the mix proportion for R2-E-100 was designed by determining the adhered mortar content to be 12% instead of 23%.
- The RAC3 was classified into R3-C, R3-E-50 and R3-E-100 according to the mix designs and RCA3 replacement ratios. As in the case of RAC2, the modified EMV design was applied for R3-E-100. The adhered mortar content was considered to be 16% instead of 32%.

For all mixtures, the water-to-cement ratio was kept constant at 0.4, and the dosage of a polycarboxyl-based plasticizer was maintained at 0.8% of the cement mass as recommended by the manufacturer. The concrete mixing and specimen preparation were carried out in accordance with ASTM C192.

### 2.4. Test Methods

**Figure 4:**
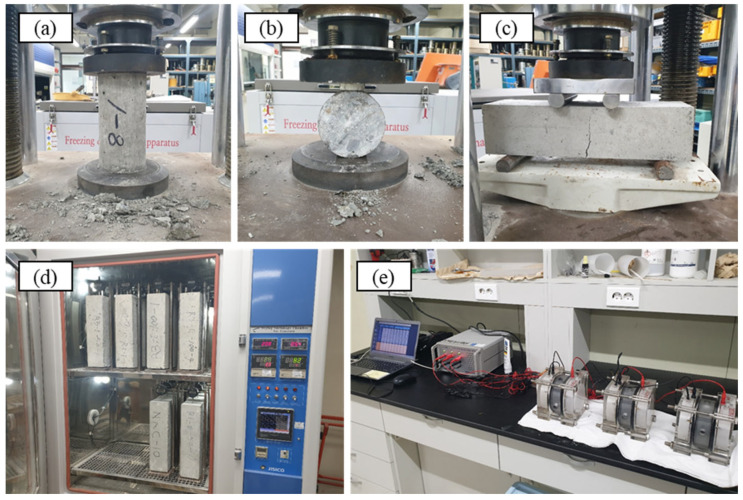
Various tests on multiple recycled aggregate concrete: (**a**) compressive strength; (**b**) splitting tensile strength; (**c**) flexural strength; (**d**) drying shrinkage; and (**e**) rapid chloride penetration test.

Workability, several physical, mechanical and durability properties of hardened concretes have been carried out. Some tests and devices used are presented in Figure 4.

The workability, a key performance indicator of fresh concrete, was determined by the slump cone test in accordance with ASTM C143.

The density and water absorption were measured to investigate the degree of porosity of concrete. The density was determined by dividing the mass in the saturated surface dry (SSD) state by the volume of the specimen. The water absorption was determined by subtracting the SSD mass by the oven-dry (OD) mass and dividing it by the OD mass.

The mechanical strength of the hardened concrete was measured using a hydraulic universal testing machine with a capacity of 1000 kN on specimens cured for 28 days. Compressive strength and splitting tensile strength were performed on cylindrical specimens with a size of 100 × 200 mm in accordance with ASTM C39 and ASTM C496, respectively. A load corresponding to a stress of 0.25 MPa/s for compressive strength and 1.0 MPa/min for splitting tensile strength was applied at a constant rate until failure (Figure 4a,b). Flexural strength was performed on prism specimens with dimensions of 100 × 100 × 400 mm in accordance with ASTM C78. The applied load was 1.0 MPa/min (Figure 4c).

The drying shrinkage was performed to evaluate the potential for concrete to shrink over time due to moisture loss in accordance to ASTM C157. The prism specimens with a size of 100 × 100 × 400 mm were mounted on a device with an electronic dial gauge after 24 h of curing, and placed in a chamber with controlled temperature and relative humidity set to 23 °C and 50%, respectively, for 100 days (Figure 4d).

The rapid chloride penetration test was performed according to ASTM C1202 to evaluate the resistance of concrete to chloride ions. Concrete disk samples with a size of 100 × 50 mm for testing were obtained by cutting the center of 100 × 200 mm cylindrical specimen, and the sides of the disk samples were coated with epoxy. The specimens were then conditioned according to the procedures specified in the standard: the specimens were placed in a vacuum desiccator at an absolute pressure of 6.65 kPa for 3 h, and water was put through the water valve to soak the specimens in water for 1 h under vacuum. The vacuum was then removed by opening the air valve and left in the water for 18 h. The conditioned specimens were assembled in a cell and applied at 60 V for 6 h (Figure 4e).

## 3. Results and Discussion

### 3.1. Workability

**Figure 5:**
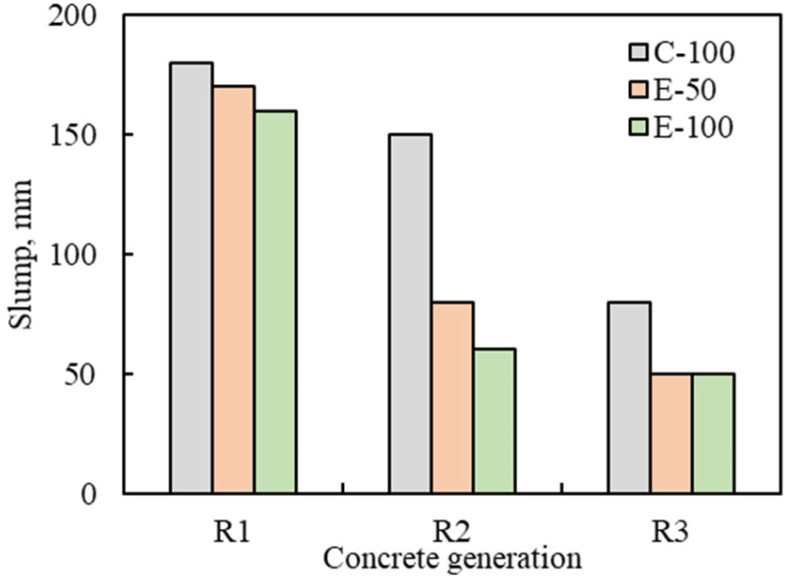
Slump of multiple recycled aggregate concrete.

The slump values of the MRAC manufactured by the CMD and the EMV method are shown in Figure 5. The slump of NAC was 180 mm, and RAC1 achieved workability nearly comparable to that of NAC, regardless of the mix designs and the RCA1 replacement ratio: the slump values of R1-C-100, R1-E-50, and R1-E-100 were 180 mm, 170 mm, and 160 mm, respectively. However, as the number of recycling cycles increased from RAC1 to RAC2 and RAC3, a decrease in slump was clearly observed, which can be explained by the relationship between the quality of RCAs and the workability of RACs. The quality of the RCAs used in this study decreased with increasing number of recycling generations. That is, the water absorption and adhered mortar content increased in the order of RCA1, RCA2 and RCA3. The water absorption of RCAs reduces concrete slump by absorbing water during mixing, thus the increased absorption, along with the number of recycling cycles, can be considered a parameter causing the slump loss of concrete. These results are consistent with the trends reported in previous studies [8,26,27,28]. The slump loss was particularly noticeable in the concretes with the EMV design, including the modified one. Compared with R1-C, the slump losses of R2-C and R3-C were 17% and 56%, respectively, while at a given recycling generation, the slump losses of E-50 concretes were 53% and 71%, and those of E-100 concretes were 62% and 69%. This slump loss occurs due to the nature of the EMV design, in which the amount of fresh mortar is reduced by the volume of the adhered mortar attached to the RCA [29,30].

### 3.2. Density and Water Absorption

**Figure 6:**
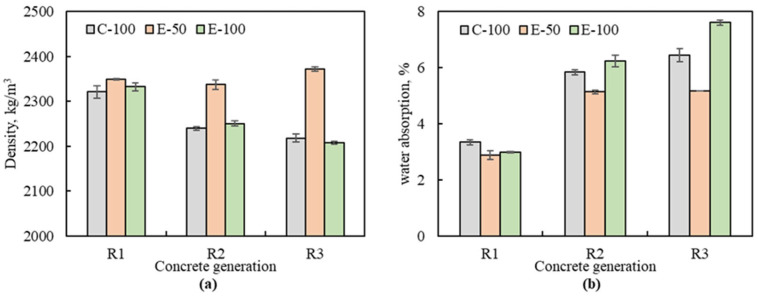
Density (**a**) and water absorption (**b**) of multiple recycled aggregate concrete.

The test results for density and water absorption are presented in Figure 6a,b. At 28 days, the density and water absorption of NAC were 2330 kg/m^3^ and 4.75%, respectively, and those of MRAC varied from 2208 to 2372 kg/m^3^ and from 2.87 to 7.61%.

The RAC1s showed little difference in density compared to NAC (≥1%). Furthermore, the densities of the RAC1s are almost similar to each other: 2321 kg/m^3^ for R1-C-100, 2349 kg/m^3^ for R1-E-50 and 2332 kg/m^3^ for R1-E-100. This is related to the quality of the aggregate used, i.e., the density of RCA1 is comparable to that of NCA.

As the number of recycling generation increases, the density of C-100 concrete gradually decreases. The densities of R2-C-100 and R3-C-100 were 3.5% and 4.4% lower than that of R1-C-100. This reduction is a result of the increased accessible porosity due to the higher adhered mortar content and lower density of RCA with increasing number of recycling [15]. Unlike the EMV method, the CMD does not consider the characteristic variation of RCA, thus the mix proportions in each recycling generation are the same. As a consequence, the total mortar volume of the C-100 concretes increases and the volume of aggregate decreases as the number of recycling cycles increases. Given that the density of mortar is usually lower than that of aggregate, the result seems reasonable. In other words, the EMV method is based on the principle that the volume of aggregate and the volume of mortar constituting unit concrete are kept constant regardless of the adhered mortar content and replacement ratio of RCA [21]. However, contradictory results were observed between the E-50 series and the E-100 series. For the E-50 concretes, the variation in density with increasing number of recycling cycles was less than 1% (2350 kg/m^3^ for RAC1, 2337 kg/m^3^ for RAC2 and 2372 kg/m^3^ for RAC3), whereas for the E-100 concretes, the density decreased by up to 5.3%. The combination of the EMV mix design with high volume and low-quality RCA often causes workability issues. Hayles et al. [31] noted that due to the lack of fresh mortar, the EMV-based concrete in the fresh state cannot be compacted properly as the cement paste does not sufficiently cover the aggregate, and this consequently increases the air content, thereby lowering the density of the concrete. Similarly, Kim at el. [17] reported that it was not possible to mold specimens of the EMV-based concrete with 100% RCA. This explains why the density of the E-100 concretes is lower than that of E-50 concretes.

Figure 6b shows the results of water absorption. The lowest and highest water absorption were observed in R1-E-50 and R3-E-100. Due to the effect of high-quality RCA1, the water absorption of C-100, E-50, and E-100 in the first generation were 3.34%, 2.87%, and 2.99%, respectively, with a difference of only 0.47% between the maximum and minimum values. However, in the second generation, the water absorption of each concrete increased to 5.84%, 5.13% and 6.24%, and the difference between the maximum and minimum values began to widen to 1.11% compared to that of the first generation concretes. In the third generation, the water absorption of C-100 and E-100 further increased to 6.44% and 7.61%, while that of E-50 tended to stabilize at 5.17% compared to 5.13% in the second generation. The difference between the maximum and minimum values was 2.44%, the largest compared to previous recycling generations. The results of water absorption are inversely proportional to those of density.

### 3.3. Mechanical Strength

**Figure 7:**
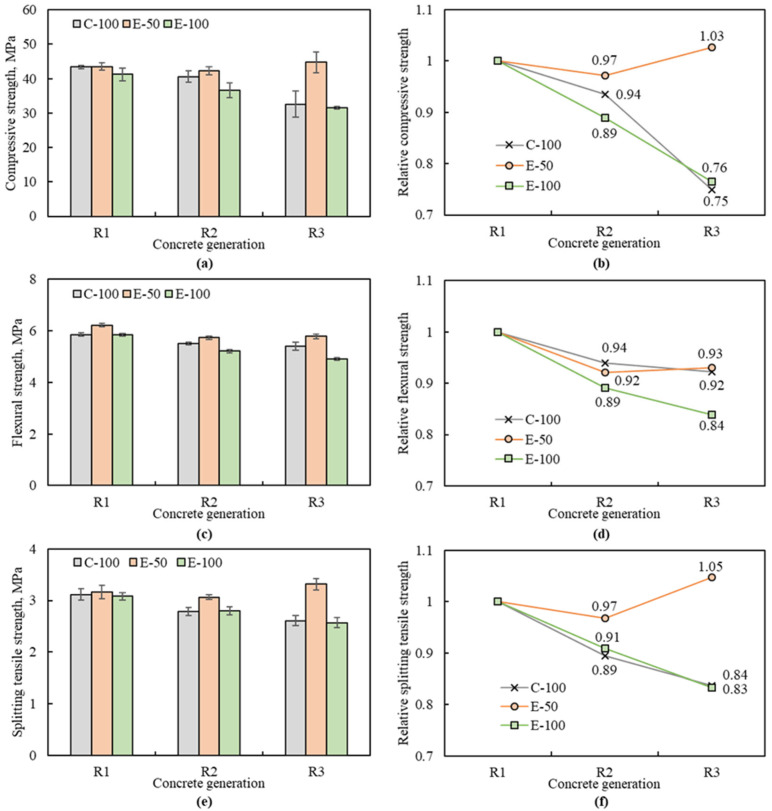
Mechanical strength of multiple recycled aggregate concrete: (**a**) compressive strength; (**b**) relative compressive strength; (**c**) flexural strength; (**d**) relative flexural strength; (**e**) splitting tensile strength; (**f**) relative splitting tensile strength.

The test results of the 28-day compressive strength, one of the most decisive properties of hardened concrete, are shown in Figure 7a,b. The compressive strength of NAC was 39.1 MPa, and that of the RACs varied from 31.5 MPa to 44.7 MPa.

The compressive strength of the first generation concretes, R1-C-100, R1-E-50 and R1-E-100, increased by 5% to 14% over that of NAC. This increase can be attributed to the quality of RCA1, which has low water absorption and high density. Concrete made with high-quality RCA exhibits comparable to or even better strength than concrete with NCA [27,28]. Specifically, the compressive strengths of the R1 concretes were similar at 43.4 MPa, 43.5 MPa, and 41.2 MPa, respectively, indicating that the influences of the mix designs and RCA replacement ratios were insignificant. This can be explained by the fact that the adhered mortar content of RCA1 (11%) is lower than that reported in the literature (about 25–55%) [27]; thus, the mix proportions do not differ significantly between the concretes designed by the CMD and the EMV method, resulting in similar strength.

The effects of the mix design methods are noticeably observed in RAC2 and RAC3. For the concrete with CMD, the compressive strength progressively decreases as the number of recycling cycles increases, as reported in previous studies [7,15]. The compressive strength of R2-C-100 and R3-C-100 was reduced by 6% and 25%, respectively, compared to R1-C-100 (Figure 7b). In contrast, for the EMV-based concretes, contradictory results were shown depending on the RCA replacement ratio. The compressive strength of E-100 concretes decreased with recycling number as observed for the C-100 concretes, with losses of 11% and 24% for the R2-E-100 and R3-E-100, respectively. However, for the E-50 concretes, the compressive strength of R2-E-50 decreased by only 3% compared to the R1-E-50, but that of R3-E-50 increased by 3%, showing that the number of recycling cycles has little effect on the E-50 concretes. The trend detected in the compressive strength results was also observed in the flexural strength (Figure 7c,d) and splitting tensile strength (Figure 7e,f). Namely, the C-100 and E-100 concretes showed a clear decrease in flexural and splitting tensile strength with increasing number of recycling cycles. From RAC1 to RAC3, the flexural and splitting tensile strength of the C-100 concretes were reduced by 8% and 16%, respectively, and those of E-100 concretes decreased by 17% and 16%. However, for the E-50 concretes, no consistent decreasing trend with the number of recycling cycles was observed. Compared to R1-E-50, the flexural strength decreased by 8% and 7% for R2-E-50 and R3-E-50, respectively, and the splitting tensile strength was reduced by 3% for R2-E-50, but increased by 5% for R3-E-50.

The gradual decrease in mechanical strength observed in the CMD-based concretes is associated with changes in RCA characteristics. The RCAs experienced deterioration in quality, such as increased water absorption and decreased density, with the number of recycling cycles. This leads to a weaker ITZ between the RCA and cement paste, resulting in strength loss [8]. In contrast, several previous studies [23,32] have reported that the EMV-based concretes have higher mechanical strength than CMD-based concretes with NCA or RCA. This is consistent with the experimental results of the E-50 series in this study. However, the strength of the E-100 series was significantly lower than that of the E-50 series, even though the same mix design method was applied. This is because the E-50 concretes made of a combination of 50% NCA and 50% RCA have a smaller ITZ between the RCA and cement paste than C-100 and E-100, and thus have stronger resistance to external forces. In fact, Fathifazl et al. [22] stated that the total mortar volume in EMV concrete is not a determining factor for strength development. In addition, the shortage of fresh mortar causes poor workability, which is responsible for the density decrease and water absorption increase in the E-100 concretes. Nevertheless, it is noteworthy that the E-100 concretes showed nearly similar compressive strength to the C-100 concrete in the third generation and splitting tensile strengths in the second and third generation, despite containing 19% and 27% less cement content.

The mechanical strength test results indicate that high-quality RCA can replace natural aggregates in terms of concrete strength without any significant barriers. Furthermore, by combining the proper RCA replacement ratio and concrete mix design, strength loss caused by multiple recycling can be prevented.

### 3.4. Drying Shrinkage

**Figure 8:**
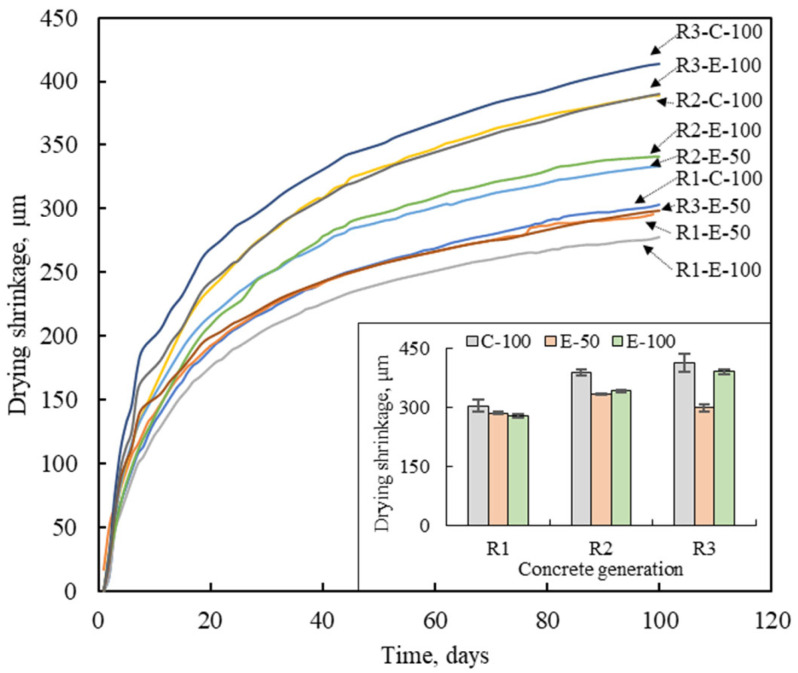
Drying shrinkage of multiple recycled aggregate concretes.

The results of drying shrinkage on various concretes for 100 days are plotted in Figure 8. For all MRACs, drying shrinkage increased in a time-dependent manner. At 100 days, the NAC exhibited drying shrinkage of 313 μm, while the MRAC exhibited a range of 278 μm to 414 μm.

The drying shrinkage of the CMD-based concrete increased with the number of recycling cycles. Compared to R1-C-100, the drying shrinkage of R2-C-100 and R3-C-100 was 28.0% and 36.2% higher, respectively. This increase can be attributed to changes in the physical characteristics of the RCA as the number of recycling cycles increases. Previous studies [27,33] have found that the quality of RCA is one of the parameters affecting drying shrinkage of concrete. In general, high-quality RCA has less effect on concrete drying shrinkage than low-quality RCA. As the number of recycling increased, the RCA experienced a decline in quality with a progressive increase in adhered mortar content. The increase in the adhered mortar content lowers the stiffness of RCA and consequently makes concrete more susceptible to drying shrinkage deformation. The drying shrinkage results of the CMD-based concretes are consistent with those of a previous study [34]. At 100 days, compared to R1-E-50, the drying shrinkage of R2-E-50 increased by 16.8%, while that of R3-E-50 increased by 10.4%, showing 8.9% less shrinkage than R2-E-50. In addition, as shown in the bar graph in Figure 8, the effect of the number of recycling cycles on the drying shrinkage of E-50 concrete was not as great as that of C-100 and E-100 concretes. This may be because the volume of each raw material in the E-50 concretes is kept similar regardless of the recycling generation. Nevertheless, the E-100 series showed an increase in drying shrinkage with the number of recycling cycles. The 100-day drying shrinkage of R2-E-100 and R3-E-100 increased by 12.3% and 40.7%, respectively, compared to R1-E-100. The higher drying shrinkage of E-100 concretes can be attributed to their water absorption performance. The drying shrinkage occurs when moisture in the pores of concrete evaporates. Due to the higher water absorption capacity than that of R-E-50 concrete, R-E-100 concrete shrinks more in dry environments. In each recycling generation, the CMD-based concrete was determined to be more shrinkable than the EMV-based concrete. Specifically, as described in previous sections, due to the high quality of RCA1, the effect of mix design method and RCA replacement ratio on the drying shrinkage of the first generation concrete was not significant. However, their influence was prominent in the second and third generations. Compared to R2-C-100, the drying shrinkage of R2-E-50 and R2-E-100 decreased by 14.2% and 12.2%, respectively. Compared to R3-C-100, the R3-E-50 and R3-E-100 showed 27.9% and 5.6% lower shrinkage, respectively. The cement content is recognized as one of the key factors that determines drying shrinkage. In light of this, it is noteworthy that the EMV-based concrete with a low total mortar volume can effectively suppress drying shrinkage [35]. Yang and Lim [30] reported that for the EMV-based concrete, the drying shrinkage decreased with lower unit cement content when the water-cement ratio was the same.

### 3.5. Rapid Chloride Penetration Resistance

**Figure 9:**
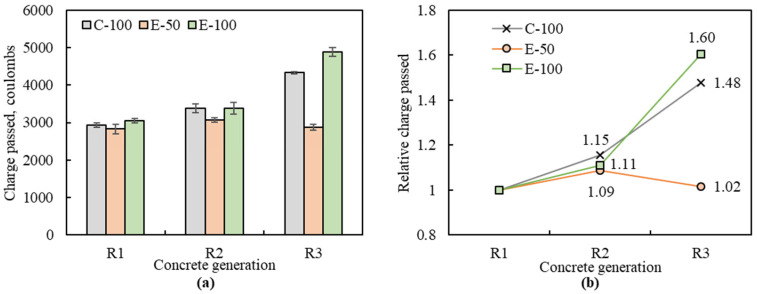
Electric conductivity of multiple recycled aggregate concretes: (**a**) electric charge passed; (**b**) relative electric charge passed.

The results of electrical conductivity tests performed on each concrete are shown in Figure 9. Based on the amount of charge passed in coulomb, chloride ion permeability can be classified into five groups, and a higher value means a lower resistance to chloride ion penetration: negligible (<100 C); very low (100–1000 C); low (1000–2000 C); moderate (2000–4000 C); high (>4000 C). The charge passed for NAC was 3423 C, indicating a ‘moderate’ level of chloride permeability. The charge passed for MRAC ranged from 2826 C to 4878 C, which put most MRAC in the ‘moderate’ group, but R3-C-100 and R3-E-100 exceeded 4000 C, indicating a ‘high’ chloride permeability.

Figure 9b shows the relative amount of charge passed during a total of three recycling of C-100, E-50 and E-100 concretes. Compared to the first generation, the charge of C-100 concretes increased by 15.4% and 47.7% in the second and third generations, respectively, indicating that the resistance to chloride penetration weakened with increasing number of recycling. This is related to the pore system of concrete, where the pores in the concrete matrix act as pathways to facilitate the movement of chloride ions [36]. With each generation, the increased adhered mortar content contains more microcracks and porosity, enabling more charges to pass through the concrete. Similar experimental results were reported by Zhu et al. [10]. For the EMV-based concretes, the amount of charge passed did not significantly change with recycling generations at 50% RCA replacement. To be specific, the amount of charge increased by only 8.6% and 1.7% in the second and third generations, respectively. This low variation can be attributed to the fact that the total mortar volume of the E-50 concrete remained constant in each generation, allowing for similar charge passed, as reported by Yang and Lee [18]. In addition, the presence of NCA in EMV-based concrete can contribute significantly to improving resistance to chloride ion penetration by segmenting the pores [37]. However, at 100% replacement ratio, the charge passed increased by 11.0% and 60.4% at the given generations. This is because E-100 concrete became less dense and more porous due to poor workability caused by the lack of fresh mortar as the number of recycling cycles increased. Therefore, to ensure chloride resistance in EMV-based concrete, the appropriate replacement range of RCA needs to be considered.

### 3.6. Comparison with Previous Studies and Discussion

**Figure 10:**
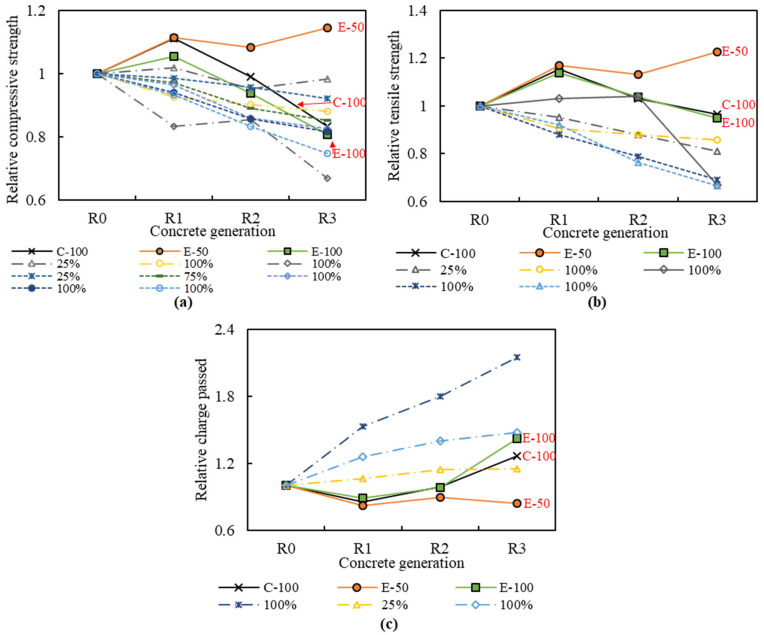
Comparison with previous studies: (**a**) compressive strength; (**b**) tensile strength; (**c**) chloride penetration [7,8,9,10,26,34].

To verify representativeness and effectiveness, the experimental data obtained in this study were compared with those of previous studies [7,8,9,10,26,34]. Figure 10 shows the variation in concrete properties as a function of the number of recycling cycles. In previous studies, a clear trend was identified where the compressive strength and tensile strength decrease and chloride penetration increases with increasing number of recycling, which is consistent with the results of the C-100 and E-100 concretes in this study. Using a lower volume of RCA (25%) could relatively compensate for the performance loss, but negative effects of multiple recycling were consistently observed [7,9,34]. On the contrary, the E-50 concrete did not exhibit a decreasing trend with the number of recycling, which demonstrates the effectiveness of the EMV method combined with a certain range of RCA replacement ratio.

**Figure 11:**
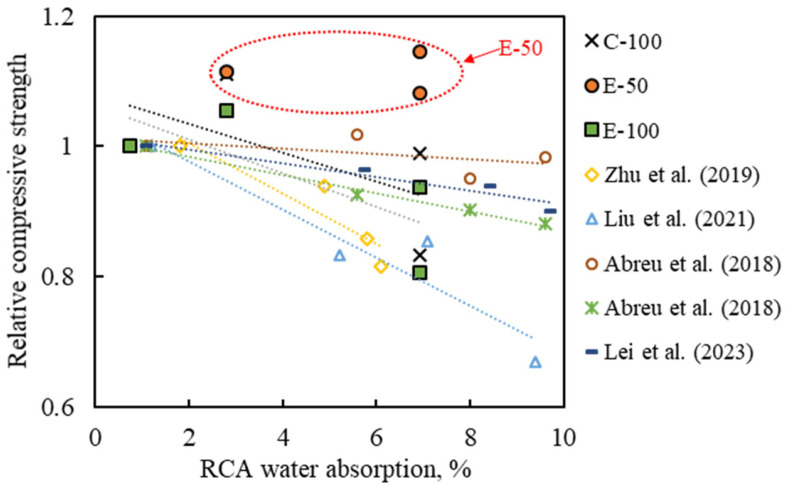
Correlation between RCA water absorption and compressive strength [7,8,10,11].

The degradation of MRAC is associated to the gradual increase in the adhered mortar content of RCA obtained from repeated recycling of concrete, resulting in RCA turning into a low-density, high-porosity material. Figure 11 illustrates the relationship between the RCA water absorption and the compressive strength of concrete presented in previous studies and in the current study. Based on the trend line plotted in Figure 11, previous studies, and C-100 and E-100 concretes show that there is an inverse relationship between RCA water absorption and the compressive strength of concrete. In contrast, the fact that E-50 concrete exhibits similar strength values regardless of RCA water absorption indicates that it is not significantly affected by aggregate characteristics. This finding is particularly important in terms of utilizing low-quality RCA without degrading concrete performance. Moreover, this can provide important implications for the use of RCA in concrete. The requirements of RCA for concrete purpose vary greatly depending on the country and institution. According to Tam et al. [1], the RILEM, German and Hong Kong standards allow a maximum water absorption of 10% for RCA for concrete, whereas in Japan, the allowable water absorption for structural and non-structural concrete is 3% and 7%, respectively. Similarly, in Korea, the water absorption of RCA for concrete should not exceed 3%. Considering these requirements, when the strict standards of the latter countries are applied, multi-recycled RCA fails to meet the current standards required for use as RCA in concrete. This indicates that low-quality RCA should only be used for low-level recycling, such as backfilling and road subbase, despite its potential to achieve good performance as in the E-50 concrete. As the production of high-quality RCA is energy-intensive, it is necessary to explore ways to promote the use of low-quality RCA through research that integrates various parameters, and to establish technical guidelines for the use of multi-recycled aggregates.

## 4. Conclusions

In this study, various properties of MRAC designed by the CMD and the EMV method were investigated.

- Concrete that includes RCA1 with low water absorption demonstrated performance comparable or superior to NAC. The slump of R1 concretes ranged within ±20 mm, and properties such as compressive strength (5–11%), tensile strength (±3%), flexural strength (14–17%), chloride resistance (11–18%), and drying shrinkage (3–12%) were improved when compared to those of NAC. This indicates that the poor performance caused by RCA in concrete compared to NCA can be offset by the use of high-quality RCA.
- As the number of recycling processes increases, the quality of RCA gradually diminishes, leading to a significant deterioration in the properties of the resulting concrete. Compared to R1-C-100 and R1-E-100, the slump, mechanical strength, and chloride resistance of R3-C-100 and R3-E-100 concretes decreased by up to 69%, 25%, and 60%, while the drying shrinkage increased by 40%. However, in contrast to this trend, the E-50 concretes exhibited a similar level of performance across three generations of recycling. Therefore, it can be concluded that the use of multi-recycled aggregate may reduce the performance of concrete, but this performance degradation can be mitigated through a combination of appropriate mix design and RCA replacement ratio.
- The experimental results in this study also indicate that RCA, even multiple RCA, have no barriers to being used as substitutes for NCA in terms of concrete performance. This could be an important finding in achieving true sustainability, enabling repeated recycling of concrete.
- Irrespective of the mix design method, remarkable slump losses were observed as the number of recycling increased, particularly in case of the EMV-based concrete. For the E-50 concrete, despite the good hardened performance, it may not suitable for building concrete unless its workability is improved. On the other hand, due to its low slump, the E-50 concrete can be utilized for prefabricated concrete elements, such as road pavement, precast structural members, sewage pipes, bricks and blocks.
- Since the concept of multiple recycling of concrete has been discussed relatively recently, there are many unknowns compared to the ‘used once’ recycled aggregate concrete. Therefore, further research is needed from various perspectives. For example, an investigation could be conducted to overcome the observed slump loss of MRAC through an increase in plasticizer dosage or through the use of supplementary cementitious materials. In addition, chemical and microstructure analyses, which have not been performed in this study, are recommended. Particularly, the analysis on the economic viability and environmental impact of multiple recycling of concrete remains unexplored, which will make a significant contribution towards achieving true sustainability in the concrete industry.

## Figures and Tables

**Table 1:** Properties of ordinary Portland cement.

Specific Gravity	Blaine Fineness, cm^2^/g	Setting Time, min		Compressive Strength, MPa			Loss of Ignition, %
		Initial	Final	3-Day	7-Day	28-Day	
3.15	3720	220	305	32.0	42.4	51.8	2.2

**Table 2:** Physical characteristics of aggregates.

Aggregate	Specific Gravity	Water Absorption, %	Adhered Mortar Content, %
NCA	2.696	0.73	-
RCA1	2.587	2.80	11
RCA2	2.400	6.92	23
RCA3	2.282	6.94	32

**Table 3:** Mixture proportions (kg/m^3^).

No.	ID	*w*/*c*	Cement	Sand	Water	NCA	RCA1	RCA2	RCA3	Plasticizer
1	NAC	0.4	410	811	164	948	0	0	0	3.28
2	R1-C		410	811	164	0	989	0	0	3.28
3	R1-E-50		388	767	155	474	532	0	0	3.10
4	R1-E-100		366	724	146	0	1065	0	0	2.93
5	R2-C		410	811	164	0	0	844	0	3.28
6	R2-E-50		348	689	139	474	0	615	0	2.78
7	R2-E-100		335	662	134	0	0	1013	0	2.68
8	R3-C		410	811	164	0	0	0	802	3.28
9	R3-E-50		315	623	126	474	0	0	697	2.52
10	R3-E-100		299	591	120	0	0	0	1128	2.39

## Data Availability

The data presented in this study are available on request from the corresponding author.
